# Assessment of photodynamic therapy efficacy against *Escherichia coli*–*Enterococcus faecalis* biofilms using optical coherence tomography

**DOI:** 10.1117/1.JBO.30.3.036003

**Published:** 2025-03-13

**Authors:** Valentin V. Demidov, Matthew C. Bond, Natalia Demidova, Ida Leah Gitajn, Carey D. Nadell, Jonathan Thomas Elliott

**Affiliations:** a Dartmouth-Hitchcock Medical Center, Department of Orthopaedics, Lebanon, New Hampshire, United States; b Dartmouth College, Geisel School of Medicine, Hanover, New Hampshire, United States; c Dartmouth College, Department of Biological Sciences, Hanover, New Hampshire, United States; d Dartmouth College, Thayer School of Engineering, Hanover, New Hampshire, United States

**Keywords:** optical coherence tomography, photodynamic therapy, biofilm, infection, orthopedic surgery

## Abstract

**Significance:**

In orthopedic trauma surgery, spatially structured biofilm ecosystems of bacteria that colonize orthopedic devices account for up to 65% of all healthcare infections, including tens of millions of people affected in the United States. These biofilm infections typically show increased resistance to antibiotics due to their structure and composition, which contributes significantly to treatment failure. Anti-biofilm approaches are needed together with clinically usable microscopic-resolution imaging techniques for treatment efficacy assessment.

**Aim:**

Antimicrobial photodynamic therapy (aPDT) has been recently proposed to combat clinically relevant biofilms (chronic wound infections, dental biofilms, etc.) using photosensitizers excited with visible light to generate reactive oxygen species that can kill bacteria residing within pathogenic biofilms. We aim to assess the efficacy of this treatment for eradication of biofilms typically present on surfaces of orthopedic devices (e.g., intramedullary nails and osseointegrated prosthetic implants).

**Approach:**

In the first phase reported here, we test aPDT *in vitro* by growing biofilms of *Escherichia coli* and *Enterococcus faecalis* bacteria (two of the seven most common pathogens found in orthopedic trauma patients) inside soft lithography-fabricated microfluidic devices. We treat these biofilms with 5-aminolevulinic acid (5-ALA)-based aPDT, evaluate treatment efficacy with optical coherence tomography, and compare with regular clinical antibiotic treatment outcomes.

**Results:**

The antibacterial efficiency of 5-ALA-based aPDT showed nonlinear dependence on the photosensitizer concentration and the light power density, with low parameters ($30\;J/{\mathrm{cm}}^{2}$ light dose, 100  mg/mL 5-ALA concentration) being significantly more effective than antibiotic-treated groups (p<0.01), reaching 99.98% of bacteria killed at $150\;J/{\mathrm{cm}}^{2}$ light dose and 200  mg/mL 5-ALA concentration setting.

**Conclusions:**

Performed experiments enable the translation of this portable treatment/imaging platform to the second phase of the study: aPDT treatment response assessment of biofilms grown on orthopedic hardware.

## 1 Introduction

Many bacteria have evolved to bind to surfaces and to each other, growing collectively in cell groups embedded within a self-produced polymer matrix of protein, polysaccharide, and deoxyribonucleic acid (DNA).^1^ These extracellular matrix-embedded cell groups, known as biofilms, are widespread in the natural environment. Biofilms are also associated with a wide range of problems in medicine, as they can colonize a wide range of tissues and implant materials, and once in place, they can be nearly impossible to eradicate with conventional antibiotics.^2^ Protected by their own architecture and internal nutrient circulation,^3^ biofilms possess a significant survival advantage, making them extremely resilient to host immune response or conventional anti-microbial therapies.

In trauma surgery, biofilm-embedded bacteria on the surfaces of orthopedic devices account for up to 65% of all healthcare bacterial infections with tens of millions of people affected in the United States.^4^^,^^5^ Current treatment strategies for this serious surgical complication include antibiotic therapy, which often fails and leads to revision surgery with implant device removal and thorough local debridement,^6^ which has high levels of associated morbidity and financial costs. Many cases result in amputation and death,^7^ because biofilms typically show 10 to 1000× higher tolerance of antibiotics^8^ compared with planktonic bacteria. The frequent ineffectiveness of antibiotics contributes significantly to treatment failure,^9^ and thus, new and alternative anti-biofilm approaches must be developed together with clinically relevant microscopic-resolution imaging techniques to accurately assess novel treatment efficacy.^10^

Antimicrobial photodynamic therapy (aPDT) has recently been proposed to combat clinically relevant biofilms (e.g., chronic wound infections, dental plaque) using photosensitizers excited with visible or near-infrared light to disrupt biofilms and kill bacteria with the resulting reactive oxygen species.^11^ Besides the independence of bactericidal effects of aPDT from antibiotic resistance patterns,^12^ the most attractive advantage of aPDT is its ability to eradicate bacteria that might otherwise be resistant to available antibiotic treatment.^13^ The efficacy of aPDT depends on the type and concentration of photosensitizer, light intensity, and duration, and the class of bacteria.^14^ Gram-positive species are often more sensitive to aPDT, whereas Gram-negative species are less susceptible due to the less effective diffusion of reactive oxygen species through their cytoplasmic membrane.^15^

Even with an effective treatment tool at hand, current imaging technology is not able to visualize biofilms associated with orthopedic implant infections.^16^ The main drawback of imaging techniques in biofilm infection diagnostics is that they often have low sensitivities and specificities, which could lead to false-positive or false-negative diagnoses.^17^ Thus, timely detection of biofilm formation on orthopedics devices (e.g., intramedullary nails and osseointegrated prosthetic implants) and intraoperative assessment of its treatment efficacy remain challenging in trauma surgery. Current diagnostics rely on clinical signs of infection such as loss of function, fever, tissue color, patient history of the predisposing condition, persisting infection, and failure of antibiotic treatment.^16^

Among the numerous laboratory imaging modalities for biofilm investigation (e.g., widefield,^18^ confocal^19^ and electron microscopies,^20^ infrared^21^ and photoacoustic spectroscopies,^22^ magnetic resonance imaging,^23^ and contrast-agent based X-ray tomography^24^), none is suitable for use in the operating room to detect orthopedic biofilms and monitor the effectiveness of their treatment. To bridge the gap into clinical relevance, we propose to use optical coherence tomography (OCT) for the detection of biofilms typically present on surfaces of orthopedic devices and routinely assess their response to treatment. Non-invasive, contrast agent-free, and amenable to *in situ* imaging, OCT has been used more and more often in recent years to characterize microbial biofilm size and morphology in a number of settings.^25^ In the first *in vitro* phase reported here, we test OCT’s ability to monitor the development of biofilms of Gram-negative *Escherichia coli* and Gram-positive *Enterococcus faecalis* bacteria inside soft lithography-fabricated microfluidic chips. We then leverage this method to assess the ability of 5-aminolevulinic acid (5-ALA)-based aPDT to destroy surface-adherent biofilms, and we compare the antimicrobial efficacy of aPDT with that of conventional antibiotics.

## 2 Materials and Methods

### 2.1 Microfluidic Model of Biofilm Growth

**Fig. 1:**
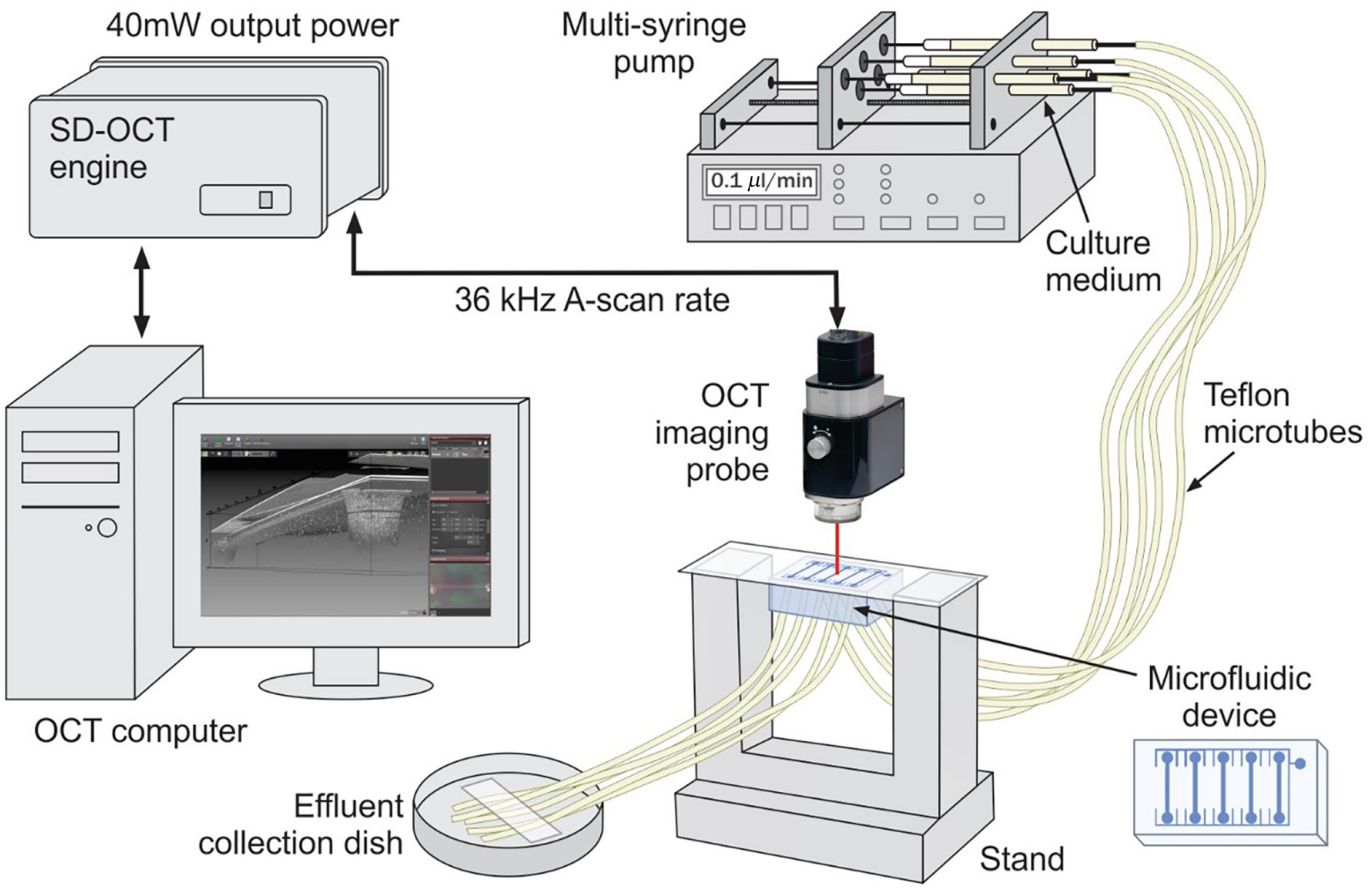
Schematic of the experimental setup.

*Escherichia coli* AR3110 and *Enterococcus faecalis* AB6 fluorescently labeled bacterial strains were cultured in 5 mL lysogeny broth overnight at 37°C at 250 rpm in an orbital shaking incubator, then pelleted, washed, and standardized prior to mixing and inoculation into microfluidic devices. In our study, we employed a versatile, yet simple microfluidic system^26^ that allowed real-time monitoring of biofilm formation in chambers and response to treatment, precise manipulation of the environmental parameters, and simultaneous multichannel experimentation while mimicking physiological conditions of biofilm formation on orthopedic implants.^27^^–^^29^ Creation of microfluidic devices is described in detail in Ref. 30. Briefly, casted poly-dimethysiloxane (PDMS, SYLGARD 184, cat. # 04019862; Dow Chemical Company, Midland, MI, United States) blocks were cut to size, hole-punched for inlet and outlet channels, and then bonded to #1.5 glass coverslips using plasma cleaning preparation of the PDMS and glass coverslips (cat. # 1152260; Azer Scientific, Morgantown, PA). As shown schematically in Fig. 1, segments of inlet tubing (PTFE #30, cat. # 06417–11; Cole Parmer, Vernon Hills, IL, United States) attached to 27 G × 1/2 needles (cat. # 305109; BD Precision, Franklin Lakes, NJ, United States) on 1 mL syringes (cat. #CMD2583; Brandzig, Spring Valley, NY, United States) were plumbed into chamber inlets, and the syringes were driven by programmable syringe pump (BS-8000; Braintree Scientific, Braintree, MA, United States). Tubing from chamber outlet channels was fed to effluent collection dishes. Each microfluidic device contained five $0.07\times 0.5\times 9\;{\mathrm{mm}}^{3}$ chambers. After 1 h of incubation, biofilms were grown for 72 h under 0.1  μL/min flow of 1% tryptone broth pumped through microtubes.

### 2.2 Imaging and Quantification

**Fig. 2:**
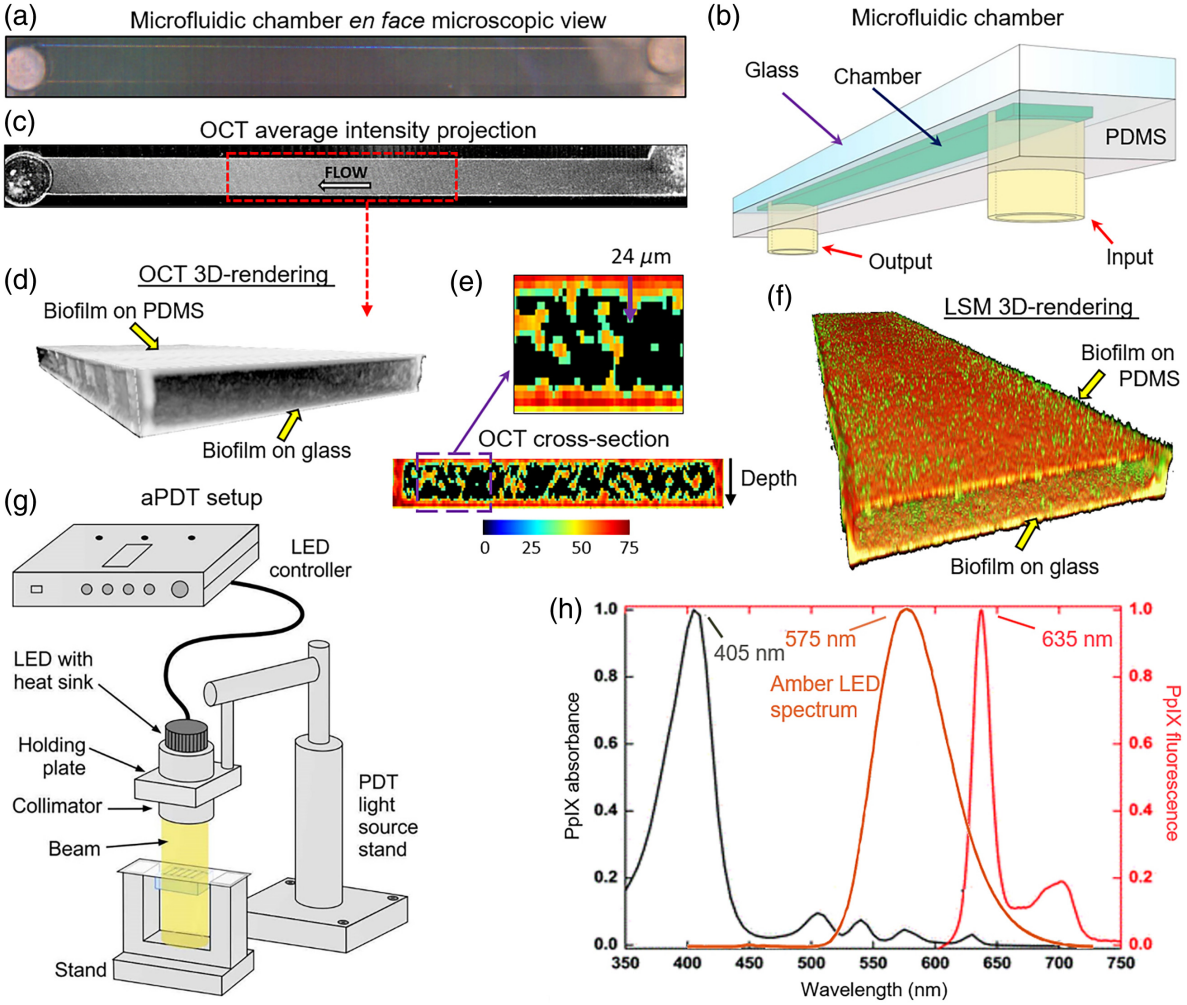
(a) Microfluidic chamber en-face microscopic view. (b) Schematic of a microfluidic chamber. The purple arrow indicates the glass attached to the PDMS, the blue arrow—microfluidic chamber, the red arrows—input and output microtube connections. (c) The average intensity projection of the OCT image of the chamber. White arrow indicates the culturing media flow. (d) OCT 3D-rendering of a 3-mm-long middle chamber segment (red-dashed rectangle in panel (c)) for analysis away from feed and drain microtube connections. Yellow arrows indicate biofilms on glass and PDMS surfaces. (e) The frontal OCT cross-section of the middle of the chamber (color bar represents OCT signal in dB). The enlarged purple rectangle-labeled area demonstrates the automatic thickness estimation of the biofilm on a glass surface equal to 24  μm at the downward-pointing purple arrow. (f) LSM 3D-rendering of a 3-mm-long middle chamber segment (red color—*E. coli*, green—*E. faecalis*). (g) Schematic of the aPDT setup. (h) PpIX excitation (black)/emission (red) and light source (amber) spectra.

Microfluidic devices were imaged with a commercial OCT system (Ganymede II, Thorlabs, Newton, NJ, United States) operating with a 36 kHz scan rate at 930 nm central wavelength. Each device was positioned on a 3D-printed stand under the OCT probe as shown in Fig. 1 to image all five chambers sequentially. To avoid strong surface reflection, the stand was tilted to 86 deg angle relative to the imaging probe. White-light microphotographs of each chamber [Fig. 2(a)] were taken prior to imaging with the camera built into the OCT probe. Three-dimensional images of each chamber shown schematically in Fig. 2(b) and containing 500×500×5000  voxels (1.4 mm deep × 1 mm wide × 10 mm long) were prepared for further processing by compensating for exponential depth-decay of the OCT signal^31^ and manually segmenting each chamber in Matlab as described in detail in Refs. 32 and 33. For visual comparison with the white-light image, the two-dimensional average intensity projection of the OCT 3D image of the same channel is shown in Fig. 2(c). The projection demonstrates the biofilm spread within the chamber, with bacteria evenly colonizing the middle part (used for analysis) and unevenly distributing in close proximities to the input and output.

Biovolume and biofilm thickness were calculated using 1500 OCT cross-sections from the 3-mm-long middle segment [red dashed rectangle in Fig. 2(c)] of each microfluidic chamber by setting a 10% threshold to cut off the noise originating from the flowing medium. The middle segment of the 9-mm-long chamber was chosen for imaging to avoid potential inconsistencies in biofilm formation near the inlet and outlet microtubes. Both biofilm-embedded and planktonic bacteria were included in biovolume quantification within the chosen chamber section shown in grayscale in Fig. 2(d) and its central cross-section in color scale in Fig. 2(e). As a secondary measure of biofilm development on the glass surface, its average thickness was calculated for each chamber. For this, an iterative algorithm was developed as schematically shown by a downward-pointing purple arrow in the enlarged cross-section area in Fig. 2(e), proceeding along each OCT A-scan from top to bottom and hard stopping at depths where the OCT signal turned down to zero. As bacteria colonies preferentially formed the most sustainable biofilms on the glass surface than on PDMS, thickness quantification on the glass surface was considered the most accurate. The described metrics were used to quantify biofilm growth and changes caused by photodynamic and antibiotic therapies.

Laser scanning microscopy (LSM) was performed for validation of OCT findings before and 24 h after aPDT treatment. For this, a Zeiss 880 line-scanning confocal microscope (Carl Zeiss Microscopy, Danvers, MA, United States) with 20× objective was used with the standard mGFP (488/510 excitation/emission) setting for *E. faecalis* and mKate2 (588/633 excitation/emission) setting for *E. coli*. Each device took 2.5 h to image compared with 10 min of OCT imaging. Obtained LSM images were saved using Zeiss Zen Blue software and then imported to Matlab for visualization. The LSM image of the same chamber scanned with OCT is shown in Fig. 2(f), where *E. coli* (red) together with embedded *E. faecalis* (green) form a solid biofilm at a 72-h time point.

### 2.3 Biofilm Treatment with Antibiotics and Photodynamic Therapy

At a 72-h time point, prior to aPDT treatment, biofilms were incubated in dark conditions for 1 h with 100  mg/mL or 200  mg/mL 5-ALA saline solution with 0.1  μL/min flow rate. 5-ALA was chosen as the photosensitizer precursor in this study due to its selective accumulation in bacterial cells and its efficient conversion to protoporphyrin-IX (PpIX). This selective accumulation enables targeted aPDT, which is particularly beneficial for treating biofilm-related infections. Compared with other photosensitizers, 5-ALA has a well-documented safety profile, with lower toxicity to surrounding tissues,^34^ making it a suitable candidate for clinical applications, especially in the context of orthopedic infections. During the incubation period, 5-ALA was taken up directly by bacteria, which then induced the accumulation of protoporphyrins in them.^35^ A 1-h time period was chosen as optimal for maximum PpIX production.^36^ After incubation, biofilms were treated with high ($250\;\mathrm{mW}/{\mathrm{cm}}^{2}$) or low ($50\;\mathrm{mW}/{\mathrm{cm}}^{2}$) amber light power density for 10 min as shown schematically in Fig. 2(g) to deliver 150 and $30\;J/{\mathrm{cm}}^{2}$ light doses, respectively. These light doses were selected based on previous studies^37^^–^^40^ that have demonstrated effective bacterial killing with similar fluences, light intensities, and exposure time, specifically using 5-ALA. Subsequent photosensitizer excitation led to bacterial death via photodamage to their cellular structures. Although a constant treatment time limited our ability to fully distinguish the effects of fluence and fluence rate, future studies will explore variations in these parameters to further optimize the aPDT protocol. PpIX excitation/emission and light source spectra are shown in Fig. 2(i). Amber light source with a spectrum peaked at 575 nm was chosen to excite PpIX at 525 to 650 nm range for deeper light penetration into biofilms compared with low-penetrating blue light.^41^ As OCT imaging of a chamber took less than 2 min, images of all five chambers of each device were taken with OCT 1, 24, 48, and 72 h after microfluidic device inoculation, then 24 h post-treatment.

Tobramycin-vancomycin antibiotic mixture was prepared by dissolving equal doses of each antibiotic in saline. Similar to 5-ALA incubation, an antibiotic mixture of three concentrations (150, 500, and 750  μg/mL) was pumped through each microfluidic device for 1 h with a 0.1  μL/min flow rate. These specific concentrations were chosen according to the optimal bone and muscle biofilm treatment results for the tobramycin-vancomycin 1:1 mixture.^42^

### 2.4 Statistical Analysis

Statistical analyses were performed using standard methods to assess the relationship between LSM and OCT measurements and to determine the significance between treatments. A Pearson correlation coefficient ρ was calculated to evaluate the strength of the relationship between biovolume measurements obtained from LSM and OCT images, with $R^{2}$ values representing the goodness of fit. Bland-Altman analysis was used to assess the limits of agreement between the two imaging techniques, providing insight into the degree of agreement between their measurements. To determine the statistical significance between treatments, pairwise comparisons were performed using the Wilcoxon rank-sum test. To control for multiple comparisons, the significance threshold was adjusted using the Benjamini-Hochberg procedure with a false discovery rate set at 0.05, lowering the threshold from p<0.05 to p<0.035.

## 3 Results

### 3.1 Detection of Biofilms with OCT

**Fig. 3:**
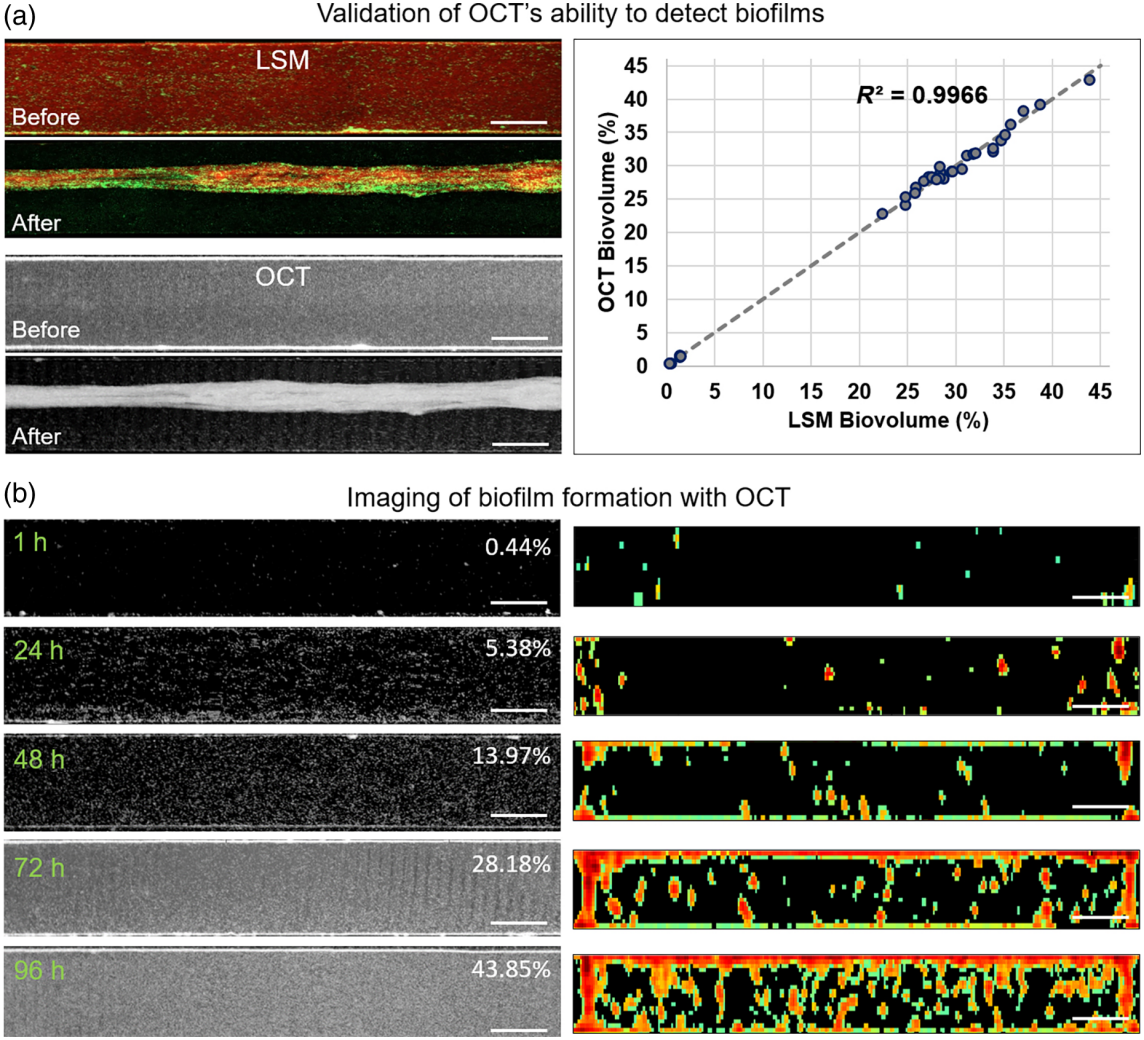
(a) Left column: Average intensity projections (AIP) of microfluidic chamber volumetric images obtained with LSM and OCT before and 24 h after antibiotic treatment. The apparent structure in the center of LSM and OCT “After” images represents a biofilm that has detached from the chamber walls and folded in response to antibiotic treatment. Scale bars are 300  μm. Right column: LSM vs. OCT biovolume correlation plot obtained from seven devices (35 data points). (b) Imaging biofilm formation with OCT. Left column: AIP of microfluidic chamber images (scale bars are 300  μm) 1 to 96 h post-inoculation, with relative biovolume shown as percentage values. Right column: corresponding medial cross-sectional images visualizing biofilms inside each chamber (scale bars are 50  μm).

Laser scanning microscopy confirmed OCT’s ability to accurately detect biofilms, which was successfully used to track their growth and quantify changes in response to antibiotic treatment and aPDT. Average intensity projections (AIP) of LSM and OCT volumetric images of a microfluidic chamber before (top row) and 24 h after (bottom row) treatment with 750 mg/mL antibiotics are shown in Fig. 3(a), left column. LSM images taken after the OCT images show nearly identical biofilm morphology, with both imaging techniques capturing very similar structural details. This close resemblance suggests a high degree of consistency between the two methods. Biovolume quantification based on data from seven devices (35 biofilms, including both controls and treated samples) revealed an exceptionally strong correlation between LSM and OCT measurements, with ρ=0.9983 (Pearson) and $R^{2}=0.9966$ for the line of equality [Fig. 3(a), right column]. In addition, Bland-Altman analysis showed limits of agreement between LSM and OCT measurements of biovolume ranging from −1.49% to 1.40%, indicating a difference of <3% between the two systems. These statistical results underscore the strong linear relationship between the biovolume data from the two techniques, highlighting the reliability and consistency of OCT for biofilm evaluation.

Fast OCT imaging enabled repetitive scanning of each microfluidic device to monitor biofilm growth. The microfluidic pump was actively pumping during imaging, but images of the central 3-mm section were acquired within 21 s; therefore, the results represent a single time point. AIP of OCT images from one of the chambers [Fig. 3(b), left column] shows attached bacteria 1 h after inoculation, with biofilm formation over the next 4 days. Image quantification shows an exponential increase in biovolume over 96 h. Corresponding medial cross-sectional images visualizing biofilms inside each chamber [Fig. 3(b), right column] reveal a gradual increase in biofilm thickness on the glass surface, reaching an average of 15  μm by the end of the monitoring period.

### 3.2 Antibacterial Efficiency of Photodynamic Therapy Compared with Antibiotic Treatment

**Fig. 4:**
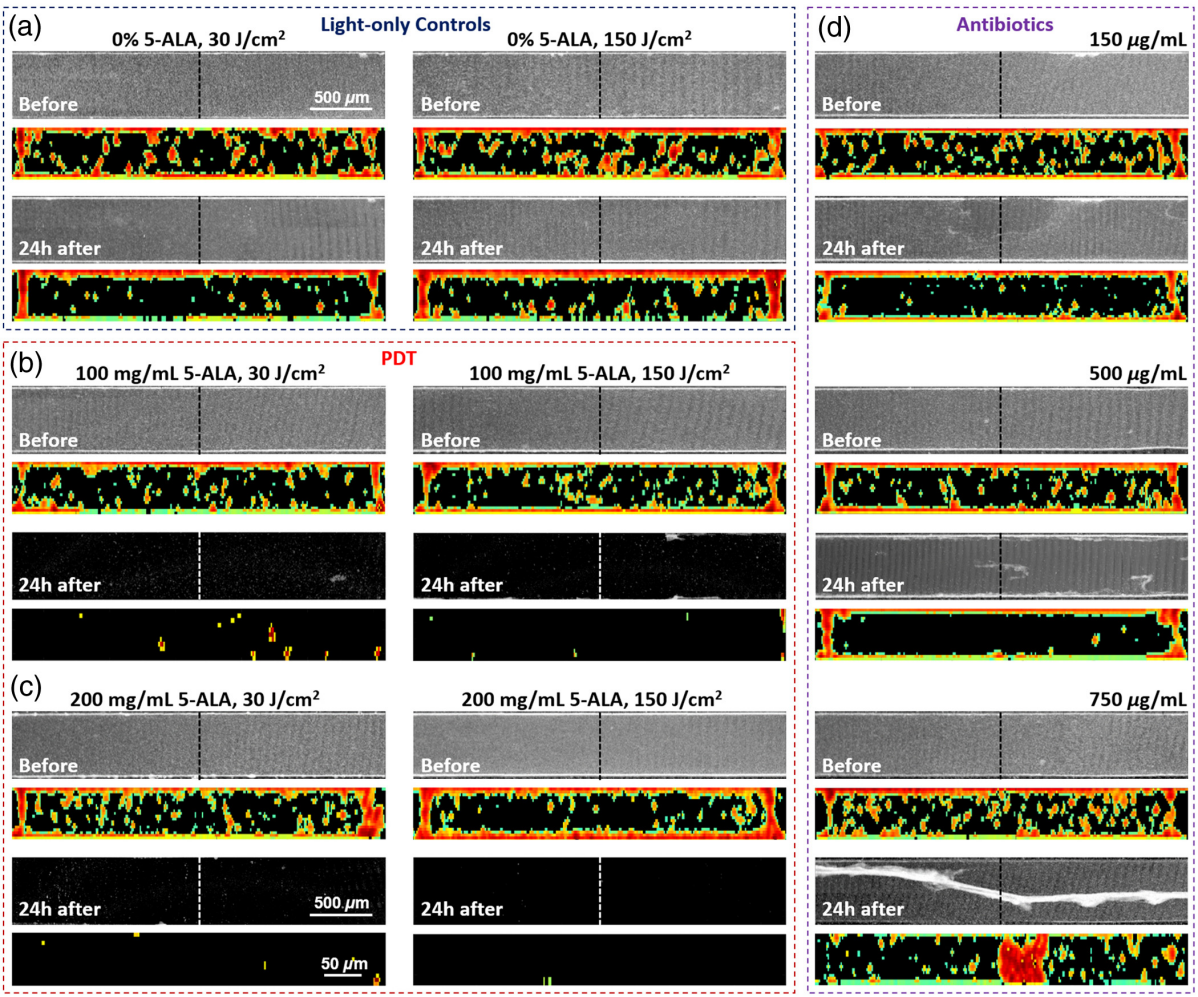
OCT AIP and medical cross-sectional images of microfluidic chambers before and 24 h after treatment: (a) Light-only controls: $30\;J/{\mathrm{cm}}^{2}$ (left) and $150\;J/{\mathrm{cm}}^{2}$ (right). Dashed lines indicate the location of corresponding cross-sections that visualize biofilms inside the chambers. (b) aPDT with 1 h of 100  mg/mL 5-ALA incubation and $30\;J/{\mathrm{cm}}^{2}$ (left) and $150\;J/{\mathrm{cm}}^{2}$ (right) light doses delivery. (c) aPDT with 1 h of 200  mg/mL 5-ALA incubation and $30\;J/{\mathrm{cm}}^{2}$ (left) and $150\;J/{\mathrm{cm}}^{2}$ (right) light doses delivery. (d) Antibiotic treatment with 1 h of tobramycin/vancomycin 1:1 mixture incubation: 150  μg/mL (top panel), 500  μg/mL (middle panel), and 750  μg/mL (bottom panel). Scale bars in all AIPs are 0.5 mm and all cross-sectional images—0.05 mm.

**Fig. 5:**
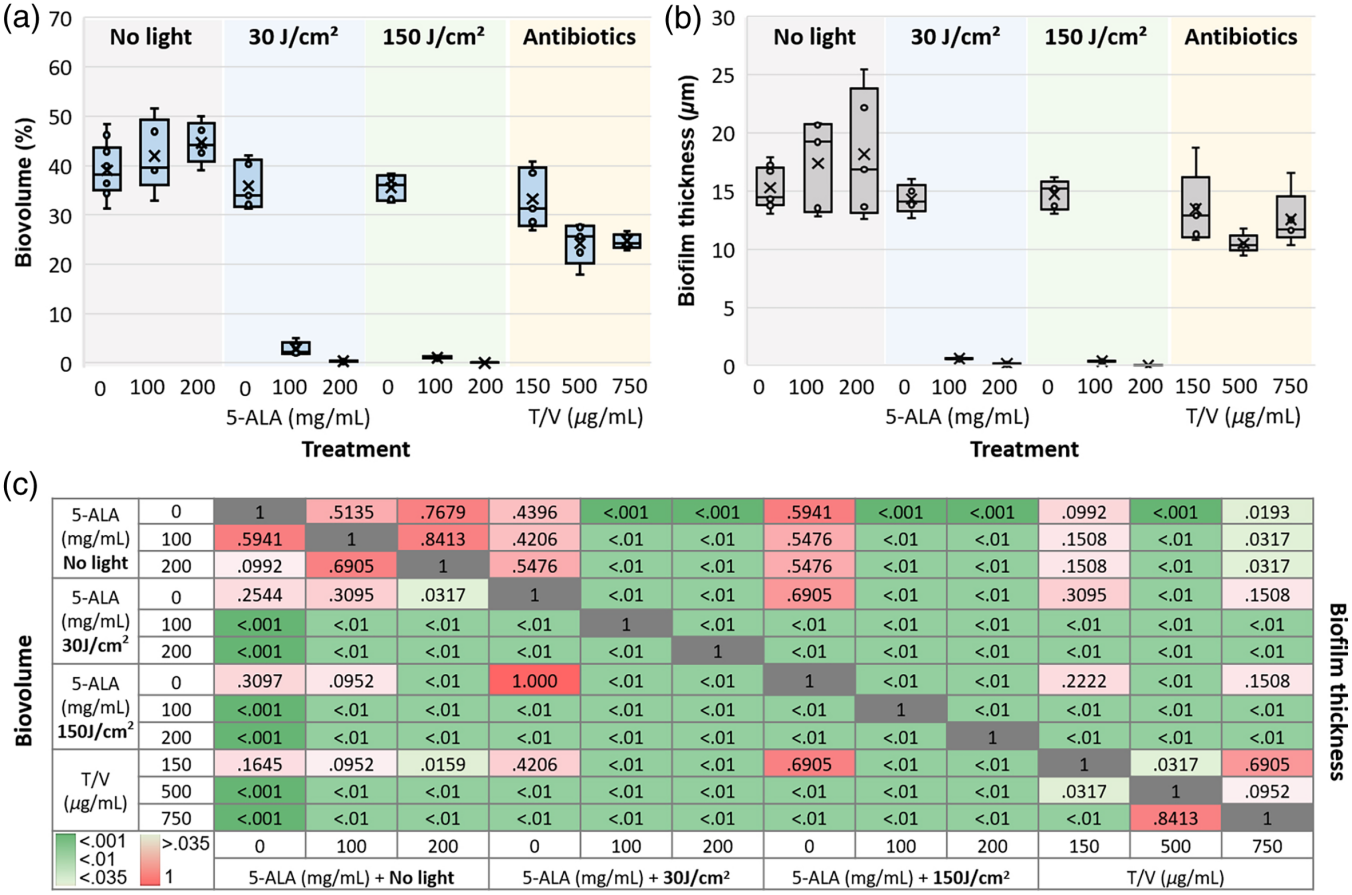
Biofilm response to treatment: (a) Relative biovolume (average biomass volume in all microchannels divided by the volume of the microchannel) 24 h after treatment with 5-ALA-only, $5\text{-}\mathrm{ALA}+30\;J/{\mathrm{cm}}^{2}$ dose, $5\text{-}\mathrm{ALA}+150\;J/{\mathrm{cm}}^{2}$, and 1 h of tobramycin-vancomycin (T/V) 1:1 mixture. (b) Biofilm thickness (average thickness of biofilms grown on the glass surface inside all microchannels) 24 h after treatment (same conditions as in panel (a)). (c) Heatmap of pairwise p-values comparing treatment groups using the Wilcoxon rank-sum test. P-values for biovolume comparison are displayed below the diagonal, and for biofilm thickness, they are shown above the diagonal. P<0.035, indicated in shades of green, represents statistically significant differences in biovolume and thickness between treatments.

Biovolume in each microfluidic chamber was quantified before treatment (baseline) and 24 h after treatment. Biofilm treatment options and results are presented in Table 1 (relative biovolume mean ± standard deviation values) and plotted in Fig. 5(a). Panels in Figs. 4(a) and 4(b) show the OCT average intensity projections of $0.07\times 0.5\times 3\;{\mathrm{mm}}^{3}$ chamber sections in the middle of the device before and 24 h after light-only treatment and 5-ALA-based aPDT. Below each projection, medial cross-sections (with locations labeled by a dashed line) visualize biofilms inside the chamber.

**Table 1:** Relative biovolume 24 h post-treatment with aPDT and antibiotics (mean ± standard deviation).

Photodynamic therapy				Antibiotic treatment	
Light dose ($J/{\mathrm{cm}}^{2}$)	5-ALA concentration			Antibiotic mixture concentration (μg/mL)	Relative biovolume
	0 mg/mL	100 mg/mL	200 mg/mL		
0	39.05 ± 5.52%	42.0 ± 7.30%	44.57 ± 4.22%	150	33.23 ± 6.13%
30	35.92 ± 4.92%	2.87 ± 1.37%	0.34 ± 0.11%	500	24.28 ± 4.19%
150	35.54 ± 2.60%	1.1 ± 0.15%	0.006 ± 0.004%	750	24.67 ± 1.48%

Both incubation with 5-ALA (no light) and light-only treatment (no 5-ALA) were found to affect the average biovolume by slowing down biofilm development, though these changes were not significantly different from controls [p>0.035, for details see statistical significance heatmap in Fig. 5(c)]. The combination of 5-ALA incubation followed by light dose delivery had a dramatic effect on biofilms. Incubation with 100 and 200  mg/mL of 5-ALA, followed by light treatment, facilitated a significant decrease in biovolume: $30\;J/{\mathrm{cm}}^{2}$ resulted in 92.7% (p<0.001) and 99.1% (p<0.001) reduction, while $150\;J/{\mathrm{cm}}^{2}$ in 97.2% (p<0.001) and 99.98% (p<0.001) reduction, respectively.

By contrast, tobramycin/vancomycin treatment had limited effect. Statistically insignificant from controls, treatment with 150  μg/mL demonstrated a 15% average biovolume reduction (p>0.1), mostly killing free-flowing bacteria as seen in Fig. 4(d), top panel. Increasing the dose to 500  μg/mL removed the majority of free-flowing bacteria but had a limited effect on the biofilm itself [Fig. 4(d), middle panel], resulting in 38% biovolume reduction (p<0.001). Raising the dose to the maximum level of 750  μg/mL caused biofilm detachment from chamber walls and folding [Fig. 4(d), bottom panel], with 37% reduction compared with controls (p<0.001), but did not significantly reduce the biovolume compared with 500  μg/mL effect (p>0.035).

Biofilm thickness changes 24 h post-treatment with aPDT and the 1:1 antibiotic mixture are presented in Table 2 (mean ± standard deviation values) and plotted in Fig. 5(b). At the 96-h time point after inoculation without any intervention, the biofilm thickness reached 15.27±1.90  μm. Similar to the biovolume measurement results, 1 h of 5-ALA incubation in 3-day-old biofilms resulted in an insignificant increase in mean biofilm thickness 24 h later, as seen in OCT cross-sectional images in Fig. 4(a) (p>0.5 for both, 100 and 200  mg/mL 5-ALA concentrations).

**Table 2:** Biofilm thickness 24 h post-treatment with aPDT and antibiotics (mean ± standard deviation).

Photodynamic therapy				Antibiotic treatment	
Light dose ($J/{\mathrm{cm}}^{2}$)	5-ALA concentration			Antibiotic mixture concentration (μg/mL)	Absolute thickness (μm)
	0 mg/mL	100 mg/mL	200 mg/mL		
0	15.27±1.90 μm	17.40±3.93 μm	18.16±5.54 μm	150	13.47 ± 3.16
30	14.33±1.28 μm	0.59±0.05 μm	0.14±0.06 μm	500	10.52 ± 0.83
150	14.72±1.29 μm	0.38±0.04 μm	0.001±0.001 μm	750	12.56 ± 2.36

Exposing biofilms to light at either low ($30\;J/{\mathrm{cm}}^{2}$) or high ($150\;J/{\mathrm{cm}}^{2}$) doses also did not significantly alter their thickness (p>0.1), although the average biovolumes slightly reduced, as shown in Table 1 and Fig. 5(a). We attribute this reduction to the decreased amount of planktonic bacteria in the analyzed volume in response to the light. Adding light treatment after 1 h of 5-ALA incubation significantly reduced the biofilm thickness, with the best effect (biofilm eradication) achieved at the high light dose and maximum 5-ALA concentration. One hour of incubation with 150  μg/mL tobramycin/vancomycin mixture did not significantly affect the biofilm thickness, as seen in before/after OCT cross-sectional images in Fig. 4(d), top panel (p>0.035). Increasing the antibiotic concentration resulted in 31.1% (500  μg/mL, p<0.001) and 17.8% (750  μg/mL, p<0.035) reduction of biofilm thickness the next day [Fig. 4(d), middle and bottom panels].

## 4 Discussion

This study demonstrates the successful application of OCT for real-time biofilm imaging and quantification, providing valuable insights into the dynamic growth and treatment response of biofilms. This imaging technology was used to track the biovolume and thickness of biofilms grown in microfluidic chambers under various treatment conditions, including aPDT with 5-ALA and antibiotic treatments. The study results highlight the potential of OCT as an efficient and non-invasive tool for monitoring biofilm development and evaluating the efficacy of antimicrobial strategies. However, this work also raises several important considerations for the broader application of OCT in biofilm research and clinical practice.

### 4.1 OCT Imaging for Biofilm Detection

The comparison between OCT and LSM revealed a high degree of consistency in biofilm morphology, as illustrated by the side-by-side image comparison in Fig. 3(a), confirming that OCT can reliably detect biofilms in microfluidic systems. The strong correlation observed between the biovolume measurements obtained from OCT and LSM ($R^{2}=0.9966$) further underscores OCT’s reliability as a tool for biofilm quantification. These results are consistent with previous studies that have highlighted OCT’s ability to accurately capture structural details of biofilms, such as thickness and volume, in a non-invasive manner.^25^ They support the utility of OCT in biofilm imaging, as it provides a less labor-intensive and more rapid alternative to traditional imaging methods such as LSM. Specifically, OCT’s ability to generate volumetric images of the biofilms in microfluidic chambers within a fraction of the time required for LSM allows for more frequent imaging, which is valuable for longitudinal studies. This high-throughput imaging is crucial for studies on the kinetics of biofilm formation and the response to different treatments, as demonstrated by biofilm growth monitoring over time [Fig. 3(b)]. Notably, the ease of setting up OCT scans and the rapid image acquisition (21 s for a 3-mm section) made it feasible to monitor biofilm formation at multiple time points without significantly disrupting the experimental system, such as the microfluidic flow.

Although OCT offers significant advantages in terms of imaging speed, its spatial resolution is lower than that of LSM, which limits its ability to resolve finer details of biofilm architecture. Despite this limitation, the utility of OCT in biofilm studies is evident, particularly in providing a comprehensive view of biofilm growth over time in a non-invasive and reproducible manner. In addition, OCT’s ability to capture real-time changes in biofilm morphology, such as detachment in response to antibiotic treatments [Fig. 4(d)], further highlights its promise as a tool for evaluating the efficacy of antimicrobial agents. Moreover, OCT’s resolution can be enhanced by optimizing the imaging probe and optics to achieve, for example, a lateral resolution of 4  μm. This is currently being explored by several research groups, including ours.^43^^,^^44^ If realized, such advancements could enable OCT to have potential applications in infection detection in clinical settings, although further research is needed before clinical deployment.

### 4.2 Antibacterial Efficiency of aPDT Compared with Antibiotic Treatment

The comparative analysis of aPDT and antibiotic treatment showed a stark contrast in their effects on biofilms. aPDT, particularly when combined with 5-ALA incubation followed by light treatment, demonstrated a remarkable ability to reduce biofilm biovolume and thickness. As illustrated in Figs. 4 and 5, the highest reduction in biovolume—up to 99.98%—was achieved with a combination of 200  mg/mL 5-ALA and a $150\;J/cm^{2}$ light dose. This substantial reduction in biovolume suggests that 5-ALA-based aPDT is highly effective in disrupting biofilm integrity, likely by inducing oxidative stress and damaging the biofilm matrix, which is supported by the literature on aPDT’s mode of action in biofilm eradication.^15^^,^^45^ The significant biovolume reduction seen here, even at low concentrations of 5-ALA, suggests that aPDT could be an effective alternative to traditional antibiotics, particularly in cases where antibiotics are less effective against biofilm-associated infections.

On the other hand, antibiotic treatment with a tobramycin-vancomycin 1:1 mixture showed limited effectiveness, with a modest reduction in biovolume and thickness. Even at the highest antibiotic concentrations tested (750  μg/mL), the treatment resulted in only a modest 37% reduction in bacterial viability, with little impact on biofilm detachment [Fig. 4(d), Table 1]. This result is consistent with the well-established challenge of treating biofilm infections with antibiotics, as the biofilm matrix can act as a physical barrier, limiting the penetration of antimicrobial agents.^46^ Furthermore, the observed partial detachment of biofilm in response to high-dose antibiotics may indicate that the biofilm structure was altered, but not entirely eradicated, suggesting that antibiotic treatments may be more effective at preventing biofilm formation than in completely disrupting pre-established biofilms. These results are in line with previous studies showing that while antibiotics can reduce the biomass of biofilms, they are often insufficient for complete eradication.^47^ Our study also demonstrates that antibiotic treatment primarily targets planktonic bacteria, as evidenced by the reduction in free-flowing bacteria, but has a limited impact on biofilm-associated bacteria. This finding emphasizes the need for more effective strategies, such as aPDT, that can penetrate the biofilm matrix and target the biofilm as a whole rather than just the planktonic component.

### 4.3 Insights into Biofilm Dynamics and Treatment Response

The combination of light and 5-ALA achieved the most significant reduction in both biofilm biovolume and thickness, with the greatest effects observed at higher 5-ALA concentrations and light doses. The results of this study, particularly the dramatic reduction in biofilm biovolume after aPDT treatment (Fig. 5), suggest that aPDT has a high potential for treating biofilm-related infections. In comparison, light-only treatments, as well as 5-ALA treatments without light, did not result in significant changes in biofilm thickness or biovolume. This suggests that while light and 5-ALA alone may affect bacterial behavior, their impact on biofilm disruption is minimal without the photodynamic reaction induced by the combination of both components. However, without the photosensitizer, the biofilm matrix itself is less affected, and this may explain the relatively modest changes in biofilm thickness observed with light-only treatments. These findings provide further evidence for the superior efficacy of aPDT in biofilm treatment compared with traditional antibiotic therapies. aPDT’s ability to disrupt biofilms more effectively than antibiotics is particularly relevant in the context of the growing issue of antibiotic resistance. Biofilms, which are often highly resistant to standard antibiotic treatment, pose a significant challenge in clinical settings, particularly in chronic infections. Therefore, exploring alternative treatment modalities like aPDT, which may offer a more effective approach to biofilm eradication, is crucial for developing new strategies to combat persistent infections.

### 4.4 Limitations of the Model and Future Directions for aPDT in Biofilm Treatment

The current study has several limitations that need to be addressed before transitioning to pre-clinical or clinical applications. First, the use of dual-species that form aggressive, well-protected biofilms^48^ in our model does not fully replicate the complexity of multispecies biofilms typically found in trauma surgery, which involve a variety of species with differing metabolic activities and biofilm-forming capabilities.^49^ The response of these multispecies biofilms to aPDT may differ, highlighting the need for further experiments using different bacterial species to better understand the optimal light spectrum and photosensitizer combinations. In addition, the photosensitizer uptake rate within thicker biofilms remains largely unexplored.^50^^,^^51^ It is likely that thicker biofilms may hinder the penetration of the drug to deeper layers, reducing treatment efficacy. The development of methods to measure diffusion rates of 5-ALA through biofilm layers, without damaging the biofilm structure as scanning electron microscopy^20^ or immunohistochemistry^52^ do, will be crucial for accurate aPDT application. Another limitation is the use of glass and PDMS surfaces in the microfluidic model, which do not fully replicate the conditions of orthopedic implants commonly colonized by biofilms in clinical settings.^5^ Therefore, future work should focus on testing aPDT on biofilms grown on materials such as titanium or stainless steel, which are more relevant to orthopedic trauma, and employing macrofluidic environments for more clinically relevant conditions. These efforts will help refine aPDT protocols and enhance their potential for real-world application.

## 5 Conclusion

Our results provide strong evidence that OCT is a powerful tool for non-invasively monitoring biofilm formation and assessing the effectiveness of various treatments. aPDT proved to be a far more effective approach for biofilm disruption compared with antibiotic treatments. Although antibiotics showed limited impact, aPDT led to substantial reductions in both biovolume and thickness, suggesting its potential as a promising alternative or adjunct to traditional antimicrobial therapies, especially in biofilm-related infections. Future research will focus on expanding the scope to include other bacteria commonly found on orthopedic implants (e.g., *S. aureus*, *S. epidermidis*, *P. aeruginosa*) and translating this aPDT treatment/imaging platform to the second phase of the study, which will assess the response of biofilms grown on orthopedic hardware. Further optimization of aPDT protocols and exploration of pre-clinical applications will be crucial to advancing this promising therapeutic approach for chronic clinical infections associated with biofilms.

## Data Availability

The datasets generated and analyzed during the current study are not publicly available but may be obtained from the corresponding author on reasonable request.
